# Associations of sitting behaviours with all-cause mortality over a 16-year follow-up: the Whitehall II study

**DOI:** 10.1093/ije/dyv191

**Published:** 2015-10-09

**Authors:** Richard M Pulsford, Emmanuel Stamatakis, Annie R Britton, Eric J Brunner, Melvyn Hillsdon

**Affiliations:** ^1^Sport and Health Sciences, College of Life and Environmental Sciences, University of Exeter, Exeter, UK,; ^2^Charles Perkins Centre, Sydney, NSW, Australia,; ^3^Exercise and Sport Sciences, University of Sydney, Sydney, NSW, Australia and; ^4^Department of Epidemiology and Public Health, University College London, London, UK

**Keywords:** Sitting, sedentary behaviour, mortality, television

## Abstract

**Background**: Sitting behaviours have been linked with increased risk of all-cause mortality independent of moderate to vigorous physical activity (MVPA). Previous studies have tended to examine single indicators of sitting or all sitting behaviours combined. This study aims to enhance the evidence base by examining the type-specific prospective associations of four different sitting behaviours as well as total sitting with the risk of all-cause mortality.

**Methods**: Participants (3720 men and 1412 women) from the Whitehall II cohort study who were free from cardiovascular disease provided information on weekly sitting time (at work, during leisure time, while watching TV, during leisure time excluding TV, and at work and during leisure time combined) and covariates in 1997–99. Cox proportional hazards models were used to investigate prospective associations between sitting time (h/week) and mortality risk. Follow-up was from date of measurement until (the earliest of) death, date of censor or July 31 2014.

**Results**: Over 81 373 person-years of follow-up (mean follow-up time 15.7 ± 2.2 years) a total of 450 deaths were recorded. No associations were observed between any of the five sitting indicators and mortality risk, either in unadjusted models or models adjusted for covariates including MVPA.

**Conclusions**: Sitting time was not associated with all-cause mortality risk. The results of this study suggest that policy makers and clinicians should be cautious about placing emphasis on sitting behaviour as a risk factor for mortality that is distinct from the effect of physical activity.

Key Messages

- Five different indicators of sitting time were not associated with mortality risk over 16 years of follow-up.
- This may be due in part to a protective effect of higher than average daily activity in this cohort.
- Previously reported relationships between sitting time and health outcomes may be due in part to low total daily energy expenditure.
- Policy makers should be cautious about recommending reductions in sitting time as a stand-alone public health intervention.
- Future studies should examine the links between sitting and mortality risk using objective methods that quantify postural allocation.

## Introduction

The health benefits of moderate to vigorous intensity physical activity (MVPA) are compelling,^1^ with inactivity estimated to cause 9% of premature mortality worldwide.^2^ Despite this, modern lifestyles are characterized by both low levels of MVPA and high levels of sedentary behaviour,^3^ i.e. sitting activities, which involve energy expenditure at resting levels (1–1.5 metabolic equivalents [METs]).^4^ Separate sitting behaviours, as well as total daily sitting time, have been linked with increased risk of all-cause^5–13^ and cause-specific^6^^,^^10^^,^^14–16^ mortality, cardiovascular disease (CVD)^17^^,^^18^ and metabolic conditions,^19–22^ independent of MVPA, indicating that sedentary behaviour is not simply the absence of physical activity but a distinct class of behaviour with its own health risks.

Previous studies have tended to focus either on selected single indicators of self-reported sitting, such as TV viewing,^12^^,^^14^^,^^16^^,^^23^^,^^24^ screen time^17^ or travelling in a car,^9^^,^^12^^,^^16^ or have only examined total sitting combined,^7^^,^^9^^,^^13^^,^^15^^,^^25^ and have observed differential associations with mortality.^11^^,^^12^^,^^14^^,^^16^ Therefore this study aims to enhance the evidence base by examining the type-specific associations of four different sitting behaviours as well as total sitting with the risk of all-cause mortality in a large cohort of UK adults with 16 years of follow-up and a wide range of covariates.

## Methods

The Whitehall II study is a longitudinal study of London-based employees of the British Civil Service. At the study’s inception in 1985, all civil servants (aged 35–55) from clerical and office support, middle-ranking executive and senior administrative grades were invited to participate and 73% consented^26^ (original sample 10 308). Baseline examination comprised a self-administered questionnaire and a clinical examination, with subsequent measurement phases alternating between a postal questionnaire alone and a postal questionnaire accompanied by a clinical examination. Approval for the study was given by the University College London research ethics committee and written consent was obtained from all participants. As sitting behaviour measures were included for the first time at Phase 5 (1997–99), this represents the baseline for the present analysis.

### Sitting time and mortality

The Phase 5 questionnaire included items on occupational and leisure-time sitting behaviours. Participants reported on average how many hours per week they spent: sitting at work including driving or commuting, and sitting at home, e.g., watching TV, sewing, working at a desk, by selecting from eight response categories (none, 1 h, 2–5, 6–10, 11–20, 21–30, 31–40, ≥ 40 h). For sitting at home, participants were given an open-text response to specify two sitting behaviours and then select a time category for each. Using the midpoint of these time categories (‘more than 40 h’ was represented as exactly 40 h), five different sitting indicators were computed: (i) work sitting (including commuting); (ii) TV viewing time; (iii) non-TV leisure time sitting; (iv) total leisure time sitting (the sum of ii and iii above); and (v) total sitting time (sum of i–iii above). Although there is no objective criterion measure of context-specific sitting, the questionnaire items used to construct the sitting exposures have demonstrated concurrent validity with past-week recalls (Pearson’s r = 0.44), activity diaries (Pearson’s r = 0.41)^27^ and have also been used in a number of previous studies where associations between sitting time and health outcomes have been observed.^12^^,^^20^^,^^21^^,^^28^

Mortality was established through the national mortality register kept by the National Health Service (NHS) Central Registry.

### Covariates

Sociodemographic covariates were age, gender, ethnicity and employment grade at phase 5. Employment grade (three levels: clerical and support, professional and executive, senior administrative grades) in the Whitehall II Study is a comprehensive marker of socioeconomic circumstance relating to social status, salary and level of responsibility.^29^ For retired participants, their last reported employment grade was considered. Health-related covariates included self-rated health (reported as excellent, very good, good, fair or poor), smoking status (current, previous, never a smoker), alcohol consumption, diet quality, body mass index (BMI) and physical functioning. Participants reported the number of ‘measures’ of spirits, ‘glasses’ of wine and ‘pints’ of beer consumed in the previous 7 days, and this was then converted to units (1 unit = 8 g) of alcohol. Diet quality was represented by frequency of fruit and vegetable consumption and was assessed using an eight-point scale from ‘seldom or never’ to ‘≥2 portions per day’. Height (m) and weight (kg) were recorded during clinical examination and BMI calculated using a standard formula. To assess perceptions of physical functioning, the SF-36 questionnaire was used and scored with the Medical Outcomes Study scoring system.^30^ The SF-36 assesses the extent to which participants’ health limits their ability to perform physical activities, ranging in intensity from vigorous (sporting and volitional exercise activities) to light (day-to-day tasks) using the responses ‘a lot’, ‘a little’ and ‘not at all’. Responses were scored, summed and transformed to scale from 0 (limited a lot in performing all types of physical activities) to 100 (able to perform all types of physical activity without limitation). This scale has been demonstrated to have high internal consistency.^31^

Physical activity covariates included daily walking time (min/day) and weekly MVPA (h/week). Physical activity was assessed using a modified version of the Minnesota leisure-time physical activity questionnaire which assesses both occupational and leisure-time activities, and which has been validated previously.^32^ Twenty items (including five open-text responses) assessed time spent engaged in walking, sports and games, gardening, housework and do-it-yourself building/maintenance projects, in hours over the previous 4-week period. Each activity was subsequently assigned an energy expenditure value in METs (where 1 MET is equal to energy expenditure at rest) using a compendium of activity energy expenditures.^33^ Moderate intensity activities were those eliciting an energy expenditure of 3–5.9 METs and vigorous intensity activities ≥ 6 METs. The energy expenditure of walking is dependent on walking pace and could not be determined from the Phase 5 questionnaire. Therefore, although some walking may have met the required energy expenditure, for the purposes of the present analyses walking did not contribute to MVPA, but daily walking time was included as a separate covariate.

### Statistical methods

Due to low numbers in the original eight response categories for sitting time, these were collapsed into four categories of as near equal numbers as the data would allow. Exact quartiles were not possible due to the non-normal distribution of the data.

To examine mortality risk from all causes across categories of the five sitting indicators, Cox proportional hazards models were fitted.^34^ Survival time was measured from the date of measurement at Phase 5 to death or censor (the earliest of the dates of withdrawal from the study or 31st July 2014). Hazard ratios and 95% confidence intervals were estimated for each sitting category with the shortest duration as the reference category. Proportional hazards assumptions were checked using Schoenfeld residuals and Nelson-Aelen cumulative hazards plots for analyses of associations between five sitting indicators and mortality. Schoenfeld residuals did not suggest evidence for any deviations from proportionality in any of the Cox models and this was consistent with observations from the Nelson-Aelen plots.

Cox models were adjusted for age, gender, employment grade and ethnicity (model 1) and subsequently for smoking status, alcohol consumption, fruit and vegetable consumption, BMI, physical functioning, walking time and MVPA (model 2). Wald chi-square tests were used to test for linear relationships in individual parameters and likelihood-ratio chi-square tests for non-linear relationships. Analyses were limited to those free from CVD at Phase 5.

To examine whether the associations between sitting and mortality differed between a priori defined subgroups, interaction terms were fitted for each sitting indicator with gender, age (in 10-year age groups), BMI (in categories according to World Health Organization [WHO] classifications of underweight, normal weight, overweight and obese)^35^ and physical activity (according to adherence to the Department of Health guidelines for MVPA).^36^ Likelihood-ratio tests were used to determine whether each interaction term improved the model fit.

To minimize potential confounding effects of occult disease at baseline, analyses were repeated after excluding those who died before Phase 6 (2001: 15 278 person-years of follow-up excluded), and then Phase 7 (2003–04: 27 808 person-years of follow-up excluded). In order to examine the possibility of bias due to differential loss from the original 1985 cohort, baseline age, gender, employment grade, alcohol consumption and the likelihood of being obese and of being a current smoker were compared between those who did and those who did not respond to questionnaire items relating to occupational and leisure-time sitting behaviour. Analyses were conducted in 2014 using STATA version 13.2.

## Results

The final sample consisted of 5132 participants who had complete data for sitting time and covariates. Sample characteristics are described in Table 1. Compared with those in the sample, those lost to follow-up between the study’s inception in 1985 and Phase 5 were slightly older at date of screening (0.42 years; 95% confidence interval [CI] 0.17, 0.67: *P* = 0.001), consumed slightly less alcohol (−1.19 units/week; 95% CI −0.64, −1.73: *P* < 0.001) and were more likely to be male (odds ratio [OR] 0.11; 95% CI 0.09, 0.13), obese (OR 0.04; 95% CI 0.03, 0.05) and in a higher employment grade (OR 0.05; 95% CI 0.03, 0.07) in 1985. Inclusion in the current analysis was not associated with smoking behaviour in 1985. A total of 450 deaths from all causes were recorded over 81 373 person-years of follow-up (mean follow-up time 15.7 ± 2.2 years).

**Table 1.** Subject characteristics at baseline (Phase 5; 1997–99). Data are mean ± SD unless otherwise specified

				Sitting Group (total from work and leisure time)				
				1 (*n* = 1273)		2 (*n* = 1384)	3 (*n* = 1239)	4 (*n* = 1236)
Age (years)				46.60 (5.83)		45.25 (6.02)	42.18 (5.31)	41.47 (4.99)
Male (%)				21.96		27.10	25.40	25.54
Female (%)				32.29		26.63	20.82	20.25
Ethnicity		White (%)		23.56		27.14	24.64	24.66
		Non-White (%)		45.52		24.14	15.86	14.48
BMI				25.63 (3.70)		25.64 (3.69)	25.56 (3.82)	26.02 (4.00)
Waist circumference (cm)				88.14 (11.46)		89.12 (11.31)	88.85 (11.06)	90.45 (11.69)
Weight (kg)				75.43 (12.95)		76.98 (12.75)	77.79 (12.92)	79.40 (13.70)
Walking (min/day)				44.45 (24.77)		44.17 (22.53)	41.21 (21.23)	40.65 (21.31)
MVPA (h/week)				15.09 (12.73)		15.70 (13.00)	12.97 (10.36)	12.61 (10.59)
Employment grade (%)	Administrative			18.81		26.58	27.22	27.39
	Prof/executive			26.72		27.39	22.77	23.13
	Clerical/support			43.23		26.90	16.33	13.54
Alcohol consumption (units/week)				12.49 (15.18)		13.45 (14.13)	13.91 (13.93)	15.94 (15.99)
Smoking status (%)		Never		24.36		26.28	25.60	23.76
		Ex		24.59		28.62	23.32	23.47
		Current		27.95		24.12	19.69	28.15
Self-rated health (%)		Very good		25.63		27.80	24.12	22.46
		Good		23.42		25.37	24.68	26.53
		Fair or poor		25.58		28.46	22.31	23.65

Hazard ratios and 95% confidence intervals for mortality risk and unadjusted mortality rates (per 1000 person-years) are presented in Table 2. There were no associations between any of the five sitting indicators at Phase 5 and all-cause mortality risk over the follow-up period in either model 1 or 2. In addition, no interaction effects were observed between the five sitting indicators and gender, age, adherence to public health guidelines for MVPA or BMI classification.

**Table 2.** All-cause mortality risk according to categories of sitting behaviours between Phase 5 (1997–99) and 31 July 31 2014

	Person yrs (x 1000)	*n*/Deaths	Rate/1000 person-years	Model 1 HR (95% CI)	Model 2 HR (95% CI)
**Work sitting** (h/week)					
≥0 & < 8	20.90	1338/175	8.37	1	1
≥8 & < 25	17.69	1121/110	6.21	0.93 (0.73, 1.19)	0.93 (0.73, 1.19)
≥25 & < 40	23.05	1438/80	3.47	0.80 (0.59, 1.07)	0.82 (0.66, 1.25)
≥40	16.73	1039/52	3.10	0.81 (0.57, 1.14)	0.81 (0.57, 1.14)
*P_trend_*				0.43	0.52
**TV sitting** (h/week)					
≥0 & <8	7.85	491/34	4.33	1	1
≥8 & <15	13.24	833/71	5.36	1.12 (0.75, 1.69)	1.00 (0.66, 1.51)
≥15 & <16	20.25	1276/106	5.24	1.08 (0.73, 1.59)	1.01 (0.68, 1.49)
≥16	15.81	1009/113	7.15	1.30 (0.88, 1.93)	1.13 (0.77, 1.68)
*P_trend_*				0.44	0.80
**Non-TV leisure-time sitting** (h/week)					
≥0 & <4	11.64	803/61	5.24	1	1
≥4 & <9	13.36	917/66	4.94	0.90 (0.65, 1.25)	0.96 (0.69, 1.33)
≥9 & <1 6	12.43	840/75	6.03	1.09 (0.79, 1.50)	1.12 (0.81, 1.54)
≥16	12.02	835/65	5.41	0.92 (0.66, 1.28)	0.89 (0.64, 1.24)
*P_trend_*				0.60	0.53
**Leisure-time sitting** (h/week)					
≥0 & <15	22.38	1400/103	4.60	1	1
≥15 & <18	18.35	1154/88	4.80	1.06 (0.79, 1.41)	1.06 (0.79, 1.41)
≥18 & <26	20.35	1282/104	5.11	1.03 (0.79, 1.36)	1.03 (0.78, 1.36)
≥26	18.95	1211/148	7.81	1.36 (1.05, 1.75)	1.29 (0.94, 1.67)
P*_trend_*				0.07	0.18
**Total sitting** (h/week)					
≥0 & <26	19.99	1273/152	7.60	1	1
≥26 & <41	21.78	1384/147	6.75	0.99 (0.79, 1.25)	1.05 (0.83, 1.33)
≥41 & <55	19.91	1239/68	3.41	0.70 (0.52, 0.94)	0.72 (0.54, 0.98)
≥55	19.70	1236/83	4.21	0.95 (0.72, 1.27)	0.92 (0.69, 1.22)
*P_trend_*				0.09	0.09

Model 1: adjusted for age, gender, employment grade and ethnicity. Model 2: further adjusted for smoking status, alcohol consumption, fruit and vegetable consumption, BMI, physical functioning, daily walking time and MVPA.

HR, hazard ratio.

## Discussion

The present study tested the hypothesis that sitting time would predict mortality risk independently of MVPA and associations would vary by type of sitting. Across almost 16 years of follow-up, no prospective associations were observed between five different indicators of sitting time and mortality from all causes.

The results of the current analysis are inconsistent with previous studies which have shown positive associations between all-cause mortality risk and TV viewing,^14^^,^^16^^,^^17^^,^^23^^,^^24^ sitting at work^37^ and total sitting time.^38^ One possible explanation for this is that the association between sitting and mortality is only evident for high volumes of sitting, and exposure in the current sample is insufficient. However there is no evidence for this, as the proportion of the sample who sit for long periods (> 8 h per day) is comparable to^9^ or higher than^5^^,^^13^^,^^14^ in previous studies where associations between sitting and mortality have been observed. Another possible explanation is that the absence of any associations between sitting and mortality is attributable to a protective effect of the high volumes of daily walking reported in the Whitehall II cohort. The public transport infrastructure in London is such that London-based employees are far likelier to stand (on buses and trains) or walk during their commute to work than those residing in other areas of the country.^39^ This is reflected in the mean reported daily walking time for the current sample (42.68 ± 22.60 min) which is over double the reported UK average (measured in the latter using an activity diary rather than a self-report questionnaire).^40^ A number of prospective cohort studies have demonstrated that both habitual active transport^41^ and daily walking are inversely associated with risk for mortality.^42^^,^^44^

Reported MVPA in the present sample is also very high, which is consistent with previous evidence that London-based civil servants on average are more active than the age-matched wider population.^45^ Importantly, analyses of data from the Whitehall II study has demonstrated reductions in mortality risk across categories of both moderate and vigorous physical activity.^26^ Previous prospective studies have reported that when analyses of associations between sitting and mortality are stratified by physical activity level, associations in the most active participants are attenuated.^5^^,^^6^^,^^11^^,^^25^ Kim *et al.*^11^ observed that TV viewing was associated with mortality risk only in those whose reported MVPA and light-intensity physical activity were below the sample median. Another study observed that in participants who were free from disease at baseline, sitting was only associated with mortality risk in those who reported zero minutes of weekly walking or moderate to vigorous physical activity.^9^

Total daily energy expenditure (TDEE) has been inversely associated with mortality risk,^41^^,^^46^ with one study reporting a 32% reduction in risk with a 1 standard deviation (SD) (equal to only 287 kcal/day) of increase in TDEE.^46^ Recent experimental evidence has also suggested that energy balance may be an important factor in the association between sitting and metabolic health.^47^ It is therefore possible that the higher than average energy expenditure in the current study may offer a degree of protection from any deleterious effects of high volumes of sitting.

Previously reported differential relationships between sitting in different contexts and mortality risk^11^^,^^12^^,^^48^ would logically reflect either a difference in the pattern of sitting (i.e. the duration of individual bouts and the number of interruptions when some activity was undertaken) or differences in behaviour-specific residual confounding (e.g. snacking while watching TV, or work-related stress). If the pattern of sitting rather than the overall duration is the important factor, it again follows that variation in energy expenditure rather than the posture of sitting may determine the relationship between sitting and mortality.

Strengths of the current study include the examination of mortality in a large sample who were regularly assessed over a substantial follow-up period, and statistical adjustment for a broad range of potential confounding factors. Detailed information on habitual physical activity was essential in examining the central hypothesis that sitting time represents a risk factor which acts independently of MVPA. Physical activity was assessed using 20 questionnaire items allowing the quantification of a broad range of activities. These activities were classified by intensity using reference MET values rather than perceived exertion. Only one previous study has attempted to adjust for the potentially confounding effect of limitations in physical functioning.^9^ Such limitations due to chronic pain, injury or ill health may alter an individual’s choice of leisure-time activity or even job role, which may therefore increase their reported sitting time in a variety of contexts.

A number of limitations must also be acknowledged. The Whitehall II study is an occupational cohort of white-collar workers. As such, all participants were healthy enough to be in active employment at the study’s inception. The use of a single industry sector, albeit one that includes a broad socioeconomic range,^29^ also limits the ability to generalize the findings to the general population. However, present findings remain relevant given the increasing proportion of workers in affluent societies employed in white-collar occupations.^49^

A degree of residual confounding must also be acknowledged. The sitting at work-mortality relationship may be affected not only by duration of sitting but also by work-related stress and the working environment,^50^ whereas the association with TV viewing may be influenced by increased snacking behaviour.^51^^,^^52^ Experimental evidence also suggests that a proportion of the unfavourable metabolic effects of prolonged sitting might be attributable to differences in energy balance.^47^ Such factors could not be accounted for in the present analysis.

The results of this study suggest that policy makers should be cautious about recommending sitting reductions without also recommending increases in physical activity.^36^ It is possible that previously reported relationships between sitting time and health outcomes are due to low daily energy expenditure, the best solution to which is to increase daily physical activity even at light intensities. At a general population level, habitual physical activity is only undertaken by a minority despite the well-established health benefits. Until more robust epidemiological and mechanistic evidence exists about the risks of prolonged sitting, the promotion of a physically active lifestyle should still be a priority. Nevertheless it is important to acknowledge that we were unable to comment on associations with disease incidence. With improving survival rates, high volumes of sitting could affect disease incidence without necessarily translating into increased mortality.

Although the examination of total sitting time remains important, future research should continue to separately consider the individual effects, determinants and confounding factors associated with sitting in different contexts. At present this will rely on self-report, as objective measures (which rely on the assumption that movement below a predetermined threshold represents sitting) are unable to determine posture. Even newer monitors such as the ActivPal (PAL Technologies, Glasgow, UK), which incorporate a thigh-worn inclinometer to determine postural changes, cannot differentiate between domains of sitting. The use of self-report provides this contextual information, although issues arising from misclassification of self-reported sitting remain. Inaccuracy and subsequent misclassification of sitting, if non-differential, may attenuate any true associations towards null, so it is possible that this contributed to the null findings in the current analyses. The items used in the current analyses also do not permit separate examination of weekday and weekend sitting, which may mask important differential associations.

Improvement in the technology of sedentary behaviour measurement will greatly aid the advancement of this field. Machine-learning and pattern-recognition approaches will allow objective determination of postural, type and intensity components of sitting from raw acceleration data.^53^^,^^54^ Further experimental evidence is also required to isolate the specific biological underpinnings of the previously observed negative effects of sitting, and to clarify which features of sitting (postural topography or energy expenditure) are important. Better definition and measurement of sitting as an exposure will allow a greater understanding of the associations with mortality risk and other health outcomes.

## Conclusions

The current study examined the associations between all-cause mortality and five separate sitting-time indicators. The results suggest that mortality risk is not associated with sitting time in this cohort. The findings may be due in part to a protective effect of a higher than average energy expenditure due to the habitual active transport associated with London-based employees. Further research is needed to address the uncertainties regarding the true nature of the exposure and the biological mechanisms that underpin previously observed associations between sitting time and health outcomes.

## Funding

The Whitehall II study is supported by grants from the Medical Research Council (G0902037), British Heart Foundation (RG/07/008/23674), Stroke Association, National Heart Lung and Blood Institute (5RO1 HL036310) and National Institute on Aging (5RO1AG13196 and 5RO1AG034454). This report is independent research arising partly from a Career Development Fellowship supported by the National Institute for Health Research between 2011 and 2014 (to E.S.). The views expressed in this publication are those of the authors and not necessarily those of the NHS, the National Institute for Health Research or the Department of Health. Open Access publication was funded by grants from the BHF and MRC.

**Conflict of interest:** None declared.
